# CD73, Tumor Plasticity and Immune Evasion in Solid Cancers

**DOI:** 10.3390/cancers13020177

**Published:** 2021-01-07

**Authors:** Haitang Yang, Feng Yao, Paul F. Davis, Swee T. Tan, Sean R. R. Hall

**Affiliations:** 1 Department of Thoracic Surgery, Shanghai Chest Hospital, Shanghai Jiao Tong University, Shanghai 200030, China; feng.yao@shchest.org; 2 Gillies McIndoe Research Institute, Wellington 6242, New Zealand; paul.davis@gmri.org.nz (P.F.D.); swee.tan@gmri.org.nz (S.T.T.); 3 Wellington Regional Plastic, Maxillofacial and Burns Unit, Hutt Hospital, Lower Hutt 5010, New Zealand; 4 Department of Surgery, The Royal Melbourne Hospital, The University of Melbourne, Parkville, Melbourne, Victoria 3010, Australia

**Keywords:** CD73, cancer stemness, treatment resistance, tumor plasticity, tumor differentiation, metastasis, targeted therapy, immunotherapy, drug repurposing

## Abstract

**Simple Summary:**

Tumors are ecosystems composed of cancer cells and non-tumor stroma together in a hypoxic environment often described as wounds that do not heal. Accumulating data suggest that solid tumors hijack cellular plasticity possibly to evade detection by the immune system. CD73-mediated generation of the purine nucleoside adenosine, is an important biochemical constituent of the immunosuppressive tumor microenvironment. In this review, the association between CD73 expression and features associated with cellular plasticity involving stemness, epithelial-to-mesenchymal transition and metastasis together with immune infiltration is summarized for a wide range of solid tumor types. Our analyses demonstrate that CD73 correlates with signatures associated with cellular plasticity in solid tumors. In addition, there are strong associations between CD73 expression and type of infiltrating lymphocytes. Collectively, the observations suggest a biomarker-based stratification to identify CD73-adenosinergic rich tumors may help identify patients with solid cancers who will respond to a combinatorial strategy that includes targeting CD73.

**Abstract:**

Regulatory networks controlling cellular plasticity, important during early development, can re-emerge after tissue injury and premalignant transformation. One such regulatory molecule is the cell surface ectoenzyme ecto-5′-nucleotidase that hydrolyzes the conversion of extracellular adenosine monophosphate to adenosine (eADO). Ecto-5′-nucleotidase (NT5E) or cluster of differentiation 73 (CD73), is an enzyme that is encoded by *NT5E* in humans. In normal tissue, CD73-mediated generation of eADO has important pleiotropic functions ranging from the promotion of cell growth and survival, to potent immunosuppression mediated through purinergic G protein-coupled adenosine receptors. Importantly, tumors also utilize several mechanisms mediated by CD73 to resist therapeutics and in particular, evade the host immune system, leading to undesired resistance to targeted therapy and immunotherapy. Tumor cell CD73 upregulation is associated with worse clinical outcomes in a variety of cancers. Emerging evidence indicates a link between tumor cell stemness with a limited host anti-tumor immune response. In this review, we provide an overview of a growing body of evidence supporting the pro-tumorigenic role of CD73 and adenosine signaling. We also discuss data that support a link between CD73 expression and tumor plasticity, contributing to dissemination as well as treatment resistance. Collectively, targeting CD73 may represent a novel treatment approach for solid cancers.

## 1. Introduction

Cellular plasticity represents a broad phenomenon whereby cells change their identity or state representing an important event in early development enabling proper tissue morphogenesis [1]. One of the best-studied examples of cellular plasticity is the epithelial-to-mesenchymal transition (EMT) whereby epithelial cells take on characteristics of mesenchymal cells while becoming more motile [2], which is essential during gastrulation and neural crest formation [3]. Under homeostatic conditions in adult tissues, cellular identity was originally believed to be hard-wired and non-malleable. However, it is now apparent that during periods of chronic perturbation, cellular identity is more pliant whereby cells within stressed tissues transition between cellular states, which is part of a natural adaptive process during wound repair and tissue regeneration [4].

Cellular plasticity is also recognized as a hallmark of cancer [5]. Importantly, poorly differentiated tumors reflected in the histological grade and a product of an EMT program are more prone to metastatic spread and poor prognosis, also are enriched in gene signatures associated with dedifferentiation and stemness [6,7]. Our understanding of the drivers of cellular plasticity in cancer is not well understood. While wide-scale cell-autonomous genomic alterations (e.g., coding mutations, chromosomal alterations) have long been recognized as a basis for tumor initiation [8], there is emerging evidence suggesting that non-cell-autonomous processes involving adaptive properties of the microenvironment also are critical features that contribute to tumor plasticity [9].

Cluster of differentiation 73 (CD73; encoded by *NT5E* [ecto-5′-nucleotidase]) molecule is a membrane-bound ecto-5′-nucleotidase ubiquitously expressed throughout the body [10] and is the major enzyme responsible for the generation of extracellular adenosine (eADO) via enzymatic dephosphorylation of nucleotide adenosine 5′-monophosphate (AMP) [11]. Other AMP-hydrolyzing enzymes can also be involved in the generation of eADO such as CD39 and ectonucleotide phosphodiesterase 1 (ENPP1), which have been covered by other excellent reviews [12,13]. CD73 has been shown to impart particularly important cell-type-specific functions involved in regulating tissue homeostasis. The physiological functions regulated by CD73-mediated generation of eADO are coupled to the interaction with seven-transmembrane domain, G-protein-coupled receptors adenosine receptor A1 (ADORA1), ADORA2A, ADORA2B, and ADORA3, each possessing a unique binding affinity and signal transduction mechanism (for a review see Olah and Stiles [14]). Under normal physiological conditions, eADO is present at low concentrations in tissues, including the brain. During periods of heightened metabolic stress such as that occurs during prolonged inflammation, eADO levels are greatly increased by enhanced 5′-nucleotidase CD73 to regulate immunity and inflammation, which is a necessary response to ensure tissue repair following injury [15].

There is emerging evidence suggesting that the CD73-adenosinergic signaling pathway is hijacked by tumors that arise because of prolonged periods of inflammation, resulting in suppression of immune-mediated disease control of tumors or limitations for resources and cell competitions due to a limited supply of oxygen [16]. Hypoxia and transforming growth factor-beta 1 (TGF-β1) represent two of the most prominent extrinsic features of the microenvironment in solid tumors that are responsible for initiating a transcriptional program triggering cellular plasticity in carcinoma cells in the form of developmental differentiation programs EMT and stemness [17]. Importantly, these changes in the tumor microenvironment (TME) also serve as drivers of CD73 expression, so it is not surprising that there is a possible link between tumorigenesis and increased CD73 expression. In this review, we will focus on the functional role of CD73 in solid tumors and its correlation with signatures of cellular plasticity (mRNA and epigenetic signatures together with an EMT signature) and host immune response, with a particular focus on solid tumors. In addition, we will touch upon the association between CD73 and metastasis, which is responsible for over 90% of cancer deaths. Finally, we will discuss the current state of drug discovery and drug development efforts focusing on CD73 and the potential of targeting the CD73-adenosinergic signaling pathway by drug repurposing.

## 2. CD73 and Human Cancer

**Figure 1:**
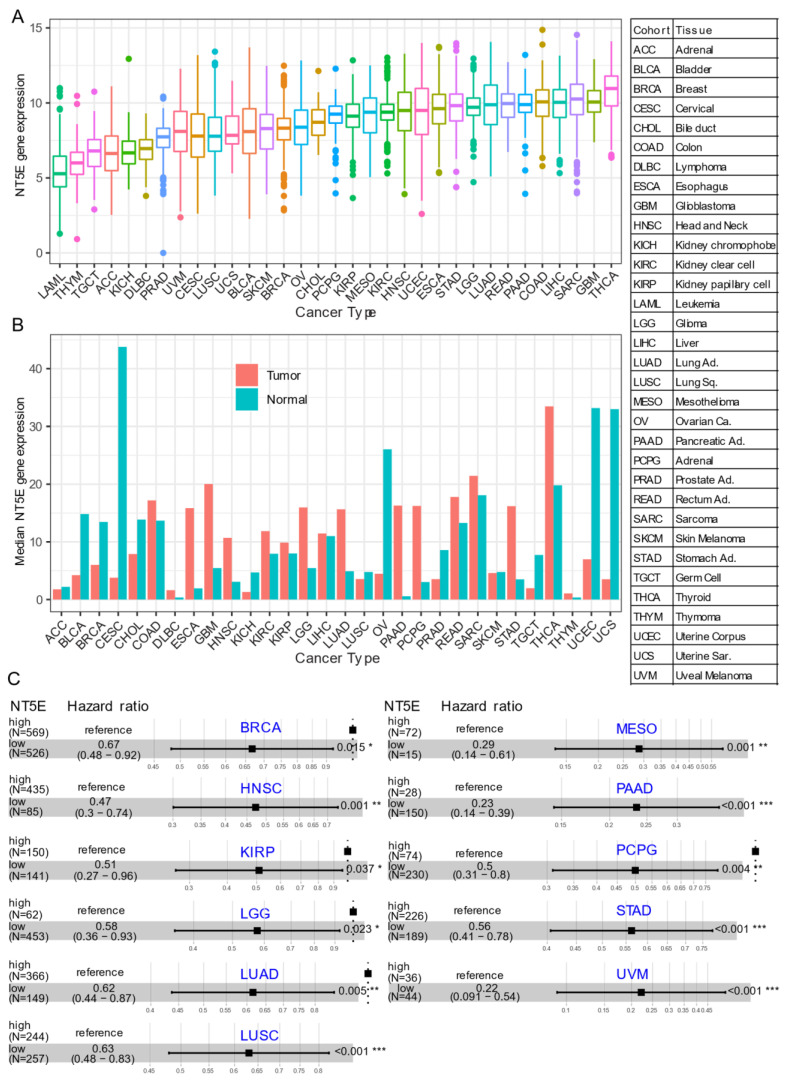
CD73 in human cancers. (**A**) *NT5E* (encoding CD73) gene expression across human solid tumors. Data were downloaded and reanalyzed from TCGA (The Cancer Genome Atlas Program) pan-cancer cohort. (**B**) Bar plot showing the *NT5E* gene expression in tumor compared with the matched normal tissue. The height of the bar represents the median expression of the indicated tumor type (red) or normal tissue (blue). (**C**) Forest blots showing the Cox proportional-hazards model-based survival analysis of cancer patients stratified by the gene expression of *NT5E* across the TCGA pan-solid cancer cohort. Only significant (*p* < 0.05) results were presented. The “high” and “low” expression groups were stratified by the optimal cutoff value using “survminer” and “survival” packages in R software. N, the total number in each group. Scale line indicates the 95% confidence interval for effect estimate for each survival-influencing factor with the hazard ratio showing to the right. ACC, adrenocortical carcinoma; BLCA, bladder urothelial carcinoma; BRCA, breast invasive carcinoma; CESC, cervical squamous cell carcinoma and endocervical adenocarcinoma; CHOL, cholangiocarcinoma; COAD, colon adenocarcinoma; ESCA, esophageal carcinoma; GBM, glioblastoma multiforme; HNSC, head and neck squamous cell carcinoma; KICH, kidney chromophobe; KIRC, kidney renal clear cell carcinoma; KIRP, kidney renal papillary cell carcinoma; LGG, brain lower grade glioma; LIHC, liver hepatocellular carcinoma; LUAD, lung adenocarcinoma; LUSC, lung squamous cell carcinoma; MESO, mesothelioma; OV, ovarian serous cystadenocarcinoma; PAAD, pancreatic adenocarcinoma; PCPG, pheochromocytoma and paraganglioma; PRAD, prostate adenocarcinoma; READ, rectum adenocarcinoma; SARC, sarcoma; SKCM, skin cutaneous melanoma; STAD, stomach adenocarcinoma; TGCT, testicular germ cell tumors; THCA, thyroid carcinoma; UCS, uterine carcinosarcoma; UCEC, uterine corpus endometrial carcinoma; UVM, uveal melanoma. Ca., carcinoma; Ad., adenocarcinoma; Sq., squamous; Sa., sarcoma. * *p* < 0.05, ** *p* < 0.01, *** *p* < 0.001. The detailed information about the bioinformatic analysis can be found in Appendix A.

Several recent studies have provided evidence that overexpression of CD73 is associated with poor patient outcomes across diverse cancer cohorts [18,19,20,21]. Gene expression analysis of the pan-cancer cohort from the public The Cancer Genome Atlas (TCGA) reveals that mRNA expression of *NT5E* is highly heterogeneous across different cancer types. For example, the most abundant expression of *NT5E* was observed in thyroid carcinoma (THCA), glioblastoma (GBM), sarcoma (SARC), and minimal in blood cancer (LAML, acute myeloid leukemia) (Figure 1A). Compared to matched normal tissue, *NT5E* is highly upregulated in the majority of solid cancer types (Figure 1B). Interestingly, this is not the case for tumors of the genitourinary system, e.g., cervical squamous cell carcinoma and endocervical adenocarcinoma (CESC), ovarian serous cystadenocarcinoma (OV), testicular germ cell tumors (TGCT), uterine corpus endometrial carcinoma (UCEC), uterine carcinosarcoma (UCS), bladder urothelial carcinoma (BLCA), kidney chromophobe (KICH) and prostate adenocarcinoma (PRAD). These observations may be due to the common embryological origin (the intermediate mesoderm) of the reproductive and urinary systems. Consistent with previous reports demonstrating the relationship between CD73 and patient survival, we demonstrate that high expression of *NT5E* predicts poor patient survival in the majority of TCGA solid tumors that have overexpressed *NT5E*/CD73 relative to paired normal tissue (Figure 1C). Collectively, the evidence provided here suggests the critical role of CD73 in a subset of non-genitourinary system-derived solid cancers and prioritizes CD73 as a potential therapeutic target in cancer.

### Regulation of CD73 Expression

Despite the detailed insights gained into the functional role of NT5E/CD73 in normal physiology and pathophysiological states, the mechanistic insights into the molecular regulation of this important nucleoside are lacking. CD73 is regulated transcriptionally through several transcription factors that bind DNA regulatory sequences within the promoter region of *NT5E* [22]. Hypoxia-inducible factor 1 alpha (HIF1α), a master regulator of cellular and systemic homeostatic response to hypoxia, binds to the *NT5E* promoter upregulating its expression. Hypoxia is also a hallmark feature of the TME [23] known to regulate *NT5E* expression and is shown to induce CD73 expression and adenosine production promoting tumor progression [24]. In triple-negative breast cancer (TNBC), both hypoxia and chemotherapy induce CD73 expression, as well as the co-expression of immune checkpoint molecules programmed death-ligand 1 (PD-L1) and CD47, which was abrogated by genetic and pharmacological blockade of HIF1α [25]. Hypoxia acting through HIF1α also drives upregulation of P1 purinergic receptors on tumor cells which are preferentially activated by adenosine [26].

Recently, the RNA binding protein poly(C) binding protein 2 (PCBP2), which is a member of a class of PCPB post-transcriptional regulatory molecules that can bind RNA and proteins [27], was shown to be upregulated in human breast cancer and associates with poor survival. Previous studies have reported that PCBP2 is upregulated in GBM [28], and hepatocellular carcinoma (LIHC) [29]. In breast cancer, RNA sequencing analysis revealed that PCBP2 regulates the expression of *NT5E* by directly binding the 3′UTR (untranslated region) and enhancing the oncogenic activity of human breast cancer cells [30]. In lung A549 adenocarcinoma cell lines and LUAD, PCBP2 was shown to bind to 5′ poly-C motif of *MYMLR*, a long non-coding RNA, regulating the transcription of MYC proto-oncogene [31]; however, whether PCBP2 also regulates *NT5E* expression in lung cancer awaits further investigation.

Methylation is a critical event in regulating the expression of genes and in the setting of cancer, the methylation levels of tumor suppressor genes or oncogenes are often deregulated. In melanoma, the promoter methylation status of CpG (5′—C—phosphate—G—3′) islands 1–7 is critically involved in regulating the expression of CD73 [32]. Methylation in *NT5E* is thought to protect against metastasis in high-risk melanomas and an inverse relationship between methylation status in *NT5E* and visceral metastasis in melanoma patients has been reported. C8161 melanoma cells, which are highly invasive and metastatic, are characterized by high mRNA expression of *NT5E* and *NT5E* CpG island remained fully unmethylated. In contrast, their isogenic parental cell line C81-61, *NT5E* CpG island was hypermethylated and *NT5E* mRNA expression was not detected. Along these lines, mitogenic and pro-inflammatory signals coming from hepatocyte growth factor (HGF) and tumor necrosis factor-alpha (TNFα), respectively, upregulate CD73 expression in melanoma cell lines that were unmethylated [33]. However, hypermethylated melanoma cell lines fail to upregulate CD73 following exposure to HGF/TNFα but do so only when pretreated prior to exposure with the demethylating agent 5-azacytidine. Importantly, the regulation of CD73 expression in unmethylated melanoma cell lines in response to mitogenic and proinflammatory signals occurs through MAPK activation of the c-Jun/AP-1 transcription complex, which binds to an intronic enhancer region in the CD73 gene. Further, upregulation of CD73 was shown in an invasive and inflammatory mouse melanoma model and is implicated in regulating phenotype switching from proliferative to an invasive mesenchymal-like state in melanoma patients who progress while on immunotherapy [33]. In human breast cancer, increased CD73 expression is inversely correlated with a lack of methylation of *NT5E* CpG island and is associated with less favorable patient outcomes [34]. In similar findings with melanoma, the methylation status of *NT5E* in breast cancer was found to associate with the acquisition of a metastatic phenotype, whereby breast tumors with unmethylated *NT5E* had a propensity to spread to viscera and brain [34]. In HPV-positive HNSCC, low methylation of *NT5E* was associated with increased CD73 mRNA expression and poor outcome [35]. Interestingly, this was not found in HPV-negative HNSCC suggesting that HPV alters the epigenome of cancer cells.

Of note, although we did not analyze the molecular genetic correlates with *NT5E* methylation and CD73 expression in human cancers, the methylation status of *NT5E* was inversely correlated with *TP53* mutation in breast cancer [34]. As well, CD73 expression was found to correlate significantly with *TP53* mutation status in patients with melanoma [36]. This has important implications, as p53 constrains cellular plasticity restricting epithelial cells from undergoing EMT [37]. Furthermore, p53 also acts as a barrier to cellular reprogramming by inhibiting expression of pluripotency-associated transcription factors and pluripotent-specific noncoding RNAs [38]. The correlation between *NT5E* expression level and mutational status also was investigated across a panel of 474 cancer cell lines from the Cancer Cell Line Encyclopedia (CCLE) [39] demonstrating a positive correlation between CD73 mRNA level and *KRAS* and *BRAF* mutational status but not *TP53* [40]. Sidders et al. [41] found no association between adenosine signaling with tumor mutational burden in the pan-cancer cohort. However, increased adenosine signaling is associated with tumors with high microsatellite instability and mutated TGF-β1 signaling, which is known to promote tumor growth via immunosuppression in addition to being a critical regulator of EMT.

Collectively, CD73 can be regulated through multiple factors. The methylation status of *NT5E* might play a role, in part, in regulating tumor plasticity and may be considered a potential independent prognostic marker in certain subtypes of cancer, which requires further independent validation. These data raise the possibility that the epigenetic regulation of *NT5E* is a critical event in tumor progression.

## 3. CD73 and Tumor Plasticity

Changes in cellular identity, known as plasticity, is critically involved in developmental and stem cell biology, and is a recognized feature in regenerative medicine, has up until recently believed to be a specialized feature endowed only to blastocyst-derived embryonic stem cells or germ cells [1]. However, there is increasing evidence suggesting that the plasticity of somatic cells may be more fluid than originally expected. This is especially the case following chronic injury/stress and cancer [1,42].

Tumor cell plasticity is a reversible program that dynamically switches among various phenotypic cellular states, such as cancer stemness, metastasis, and therapy-resistant status, which contribute to tumor relapse, treatment failure, and patient death [5,43,44]. The tumor suppressor gene p16INK4a/CDKN2A, which is functionally linked with cell-cycle regulation and senescence, has also been shown to regulate plasticity and cancer through chromatin remodeling [45]. In normal human mammary epithelial cells, repression of p16^INK4a^/CDKN2A induced the upregulation of CD73, which was subsequently used to identify a rare subset of somatic cells contained within the normal human breast tissue that give rise to functional derivatives of each germ layer when transplanted in immune-incompetent mice [46]. CD73^high^ somatic breast cells were enriched in the epithelial cell adhesion molecule EpCAM (epithelial cell adhesion molecule), as well as transcription factors associated with pluripotency SOX2, OCT3/4 and NANOG. Prior to this, Reynolds et al. (2006) demonstrated that loss of p16INK4a activity was associated with upregulation of the enhancer of zeste homolog 2 (EZH2), a polycomb group of proteins. EZH2, which is a histone methyltransferase, was shown to be causally involved in DNA hypermethylation of the transcription factor HOXA9 to promote transcriptional silencing in human mammary epithelial cells [47]. Hypermethylation of HOXA9 and repression of gene expression has been shown to promote mammary epithelial cell growth, survival, and dysregulated tissue morphogenesis [48]. Moreover, HOXA9 is known to bind to the promoter region of *BRCA1* gene and regulate its expression and reduced levels of HOXA9 and BRCA1 are associated with poorly differentiated and highly aggressive human TNBC. Thus, this evidence suggests that p16^INK4a^ is critically involved in regulating epithelial cell plasticity, whereby loss of p16^INK4A^ induces a phenotypic state driven by a specific combination of expressed genes including CD73 that enable cells to cope with stress, evade immune detection and promote invasion. The various genes responsible for binding to *NT5E* promoter regions directly regulating CD73 expression, however, remain unclear and require further investigation. Conditionally reprogrammed Oct4^high^/Klf4^high^ mouse embryonic fibroblasts (MEFs) proceed through a transition MET (mesenchymal-to-epithelial) state of CD73^high^EpCAM^high^ and then to pluripotency [49]. There is emerging evidence suggesting that tumor-derived CD73 acts as a critical mediator maintaining tumor stemness [50,51], promoting tumor metastasis [52,53,54], and facilitating tumor escape following treatment [55,56], thereby establishing a link between CD73 activity and plasticity.

### 3.1. Tumor Stemness

Cancer stemness plays a fundamental role in tumor initiation, relapse, and heterogeneity, as well as therapy resistance [57]. Recent studies provide evidence to support the role of CD73 in regulating tumor stemness in ovarian, hepatocellular, pancreatic neuroendocrine, and breast cancer [50,51,53,58,59]. The molecular mechanisms whereby CD73 promotes cancer stemness is still under investigation. Tumors are complex ecosystems consisting of differentiated cancer cells, cancer stem cells along with a complex stroma composed of mesenchymal cells (endothelial, pericytes, cancer-associated fibroblasts [CAFs]) and immune infiltrates [60]. Therefore, in addition to the influence of the cell-of-origin and oncogenic signaling pathways and their downstream targets, the feature of cancer stemness also is influenced by cell-extrinsic factors largely through epigenetic mechanisms dictated by the bidirectional communication between tumor epithelium and the microenvironment [61]. This has important implications, as CD73 is widely expressed on both tumor and non-tumor stroma, including endothelial cells, CAFs, T cells, B cells and NK cells.

A recent study by Ma et al. demonstrated a positive correlation between CD73 expression and sphere-forming capacity in vitro and in vivo in hepatocellular carcinoma [53]. In addition, CD73 positively regulates the expression of stemness-associated genes. Mechanistically, CD73 facilitates the property of cancer stemness by upregulating SOX9 expression via AKT-c-MYC signaling, and inhibiting glycogen synthase kinase 3β that leads to stabilizing its protein [53]. Their data support CD73 as a novel surface marker for identifying cells with features of cancer stem cell behavior in hepatocellular carcinoma.

**Figure 2:**
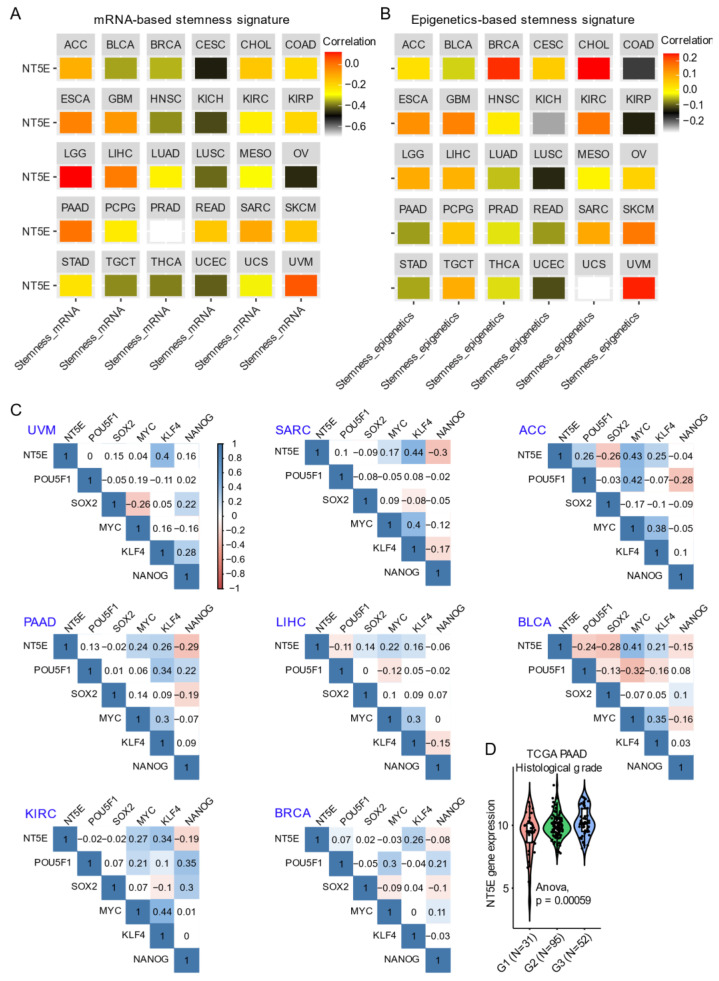
CD73 and tumor stemness signature. (**A**,**B**) Correlation analysis of the stemness signature score and *NT5E* gene expression. Curated mRNA- (**A**) and epigenetics-based (**B**) stemness scores derived by the Stemness group were used (see the methods in Appendix A). (**C**) Correlation matrix showing the correlation between gene expression of *NT5E* and four classical transcription factors (OCT-3/4, KLF4, SOX2, and c-Myc) that reprogram pluripotent stem cells across several TCGA (The Cancer Genome Atlas) solid cancer types. Positive (in blue) and negative (in red) correlations are shown to the right, with color intensity and the size of the circle proportional to the correlation coefficient. Non-significant correlation coefficient values are left blank. On the right side of the correlogram, the legend color shows the correlation coefficients and the corresponding colors. *p*-value < 0.05 is considered significant. (**D**) Violin plots showing the association between *NT5E* gene expression and histological grades of TCGA PAAD (pancreatic adenocarcinoma) tumors. Note that the information on the histological grades is only available for several cancer types. The detailed information about the bioinformatic analysis can be found in Appendix A.

Integrating CD73 gene expression from the TCGA pan-cancer cohort dataset with curated mRNA-based and epigenetically-regulated RNA expression-based stemness scores established by the Stemness group [62] show the heterogeneity in CD73 gene expression with two different algorithms-based stemness signature scores across solid cancer types (Figure 2A,B). This suggests potentially differential roles of CD73 in regulating cancer stemness across tumors originating from different lineages. For example, the strongest positive correlation between both mRNA-based and epigenetics-based stemness signature with CD73 mRNA expression was observed for UVM and LGG. Interestingly for BRCA, mRNA-based stemness signature was negatively correlated with CD73 expression, while the eEpigenetics-based stemness signature shows a strong positive correlation. The inconsistency might be due to the critical role of epigenetics in regulating and maintaining the stemness state [61,63], and implies the interaction between CD73 and epigenetic profiles. Additionally, there is a strong correlation between estrogen receptor (ER)-negative and TNBC metastatic breast cancer and CD73 overexpression related to the unmethylated status of *NT5E* [34]. Further, ER-negative and TNBC-positive breast cancer patients with unmethylated *NT5E* have poorer disease-free survival and overall survival compared with ER-positive breast cancer, suggesting a selection of unmethylated clones overexpressing *NT5E* is a critical event in breast metastasis. Interestingly, *NT5E* methylation status was inversely correlated with *TP53* mutation status while being positively correlated with ADO signaling [41].

The transcription factors POU5F1 (OCT-3/4), KLF4, SOX2, and c-MYC reprogram somatic cells to an embryonic-like state by activating pluripotency genes [64]. In adult tissues, stemness is associated with a dedifferentiated state and a feature of cellular plasticity that is also associated with cancers [65]. For example, in hepatocellular carcinoma CD73 expression regulates hepatocellular cancer stem cells via the AKT-c-MYC axis [53]. In line with this, CD73 is significantly correlated with KLF4 and c-MYC compared with POU5F1 and SOX2 across a wide-range of solid cancers (Figure 2C). A dramatic upregulation of CD73 in PAAD compared to the matched normal tissue (Figure 1B) was related to a high histological grade, a well-established indicator marking the degree of tumor differentiation (Figure 2D). In an independent PAAD dataset, CD73 expression was shown to be upregulated in both (classical and basal) subtypes of pancreatic cancer [66]. DNA methylation of CD73 was lower in tumors compared with matched normal pancreatic tissue. Further, the CD73 expression level was negatively correlated with the methylation levels of CD73 in PAAD. Hypomethylation of specific CpG loci combined with high CD73 expression was associated with poor overall survival suggesting that methylation and CD73 expression could potentially be used to better stratify patients in PAAD. Finally, elevated CD73 was also associated with an increased expression of P1 purinergic receptors A2A, A2B and A3, as well as an increase in tumor mutational burden. Likewise, Vogt et al. (2018) [35] demonstrated an inverse correlation between methylation of *NT5E* and its mRNA expression levels, which was associated with poor outcomes in HPV (human papillomavirus)-positive tumors in HNSC. Thus, it appears that the association between CD73 and indices of cancer stemness (mRNA- and epigenetic-based) may be highly context-specific.

### 3.2. CD73, EMT and Cancer Metastasis

Cancer metastasis is the major cause of cancer-related death. In addition to its enzymatic role in generating immunosuppressive eADO, CD73 also has enzyme-independent functions impacting tumor growth directly such as through interaction with extracellular matrix proteins enhancing cell adhesion, which may contribute to promoting the dissemination and metastatic spread of cancer cells. In support of this, preclinical evidence has revealed a link between CD73 and metastasis across a wide range of solid cancer types [52,53,54,67,68]. Consistently, CD73 is shown to be involved in cell-cell and cell-matrix interactions [69,70], and CD73-deficient mice exhibit resistance to experimental metastasis [71]. Despite this, the molecular mechanism whereby CD73 promotes tumor metastasis are poorly understood.

Interestingly, both tumor and host cell-derived CD73 has been shown to participate in the tumor immune escape and metastasis [53,54]. In tumor cells of hepatocellular carcinoma, adenosine produced by CD73 binds to the high-affinity adenosine A2A receptor (A2AR), which then activates Ras-proximate-1 (Rap1) [53]. Sequentially, P110β is recruited to the plasma membrane and triggers phosphatidylinositol (3,4,5)-triphosphate (PIP3) production, consequently promoting the activation of AKT-mediated tumor metastasis. CD73 is also expressed on multiple host cell types including T cells [72], endothelial cells [73], and fibroblast cells [74]. Mice with CD73 ablation substantially suppress the growth of several types of tumors and are resistant to metastasis [71,75,76], which is likely due to reactivation of host anti-tumor immunity.

**Figure 3:**
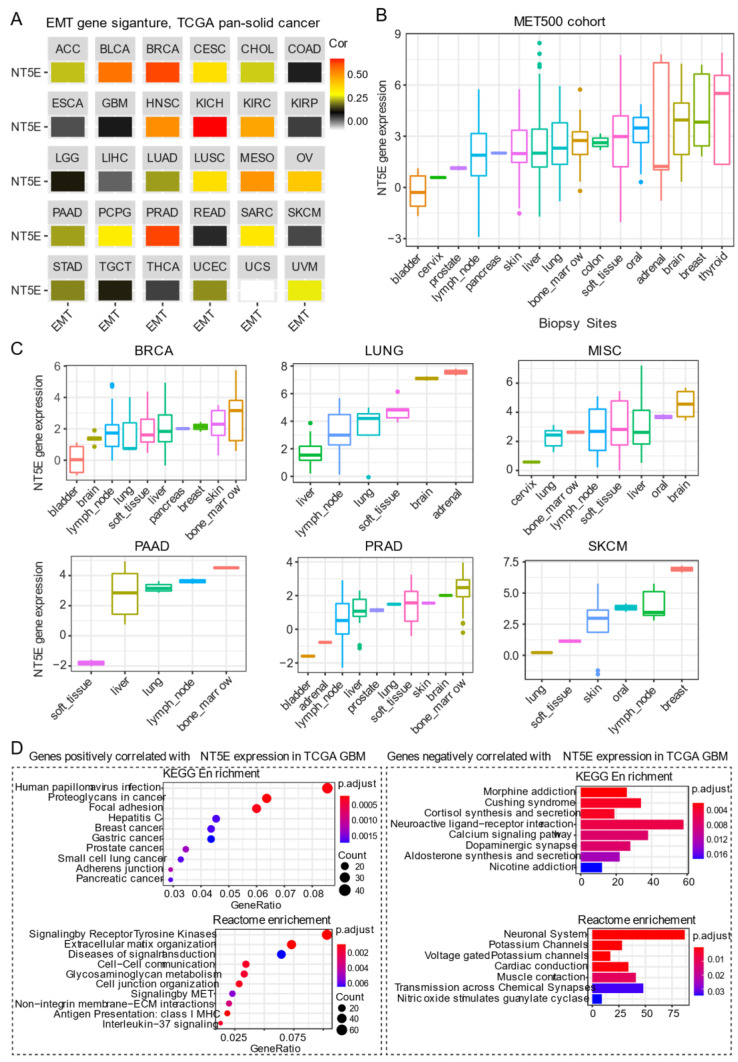
CD73 and tumor metastasis. (**A**) Correlation analysis of the epithelial-to-mesenchymal transition (EMT) and gene expression of *NT5E* across the TCGA pan-solid cancer cohort. Curated EMT signature score was used (see the methods in Appendix A). (**B**) *NT5E* gene expression across the metastatic tumors at different sites. MET500 cancer cohort was used, which provides the transcriptomic data of 500 adult patients with various metastatic solid tumors (see the methods in Appendix A). (**C**) *NT5E* gene expression across different secondary tumors with the same primary origin. (**D**) Pathway enrichment analysis of genes significantly positively (left) or negatively (right) correlated with *NT5E* gene expression in TCGA GBM (glioblastoma multiforme) tumors. The detailed information about the bioinformatic analysis can be found in the supplementary material.

The most widely studied phenomenon of phenotypic plasticity is the EMT [77], a reversible molecular program whereby epithelial cells lose apicobasal polarity while progressively acquiring mesenchymal features including cell elongation and enhanced motility. Besides its role in development, activation of EMT programs is a prominent feature in cancer whereby carcinoma cells exploit this program to promote progression and metastasis. Transitioning between these states involves a coordinated interplay between the expression of gene networks regulated by distinct chromatin landscapes. CD73 expression positively correlates with curated EMT signature score [78,79] across the majority of solid cancer types (Figure 3A). For example, a strong correlation between CD73 and EMT signature in BRCA, kidney chromophobe (KICH), kidney clear cell carcinoma (KIRC) and prostate cancer (PRAD) has been observed. Cancers with some representative squamous morphology (BLCA, CESC, HNSC, lung squamous cell carcinoma [LUSC] and mesothelioma [MESO]) also showed a positive correlation between CD73 mRNA expression and EMT signature. Not all tumors exhibited a link between CD73 expression and EMT gene signature, as found for gastrointestinal adenocarcinomas (ESCA, STAD, colon adenocarcinoma [COAD] and rectum adenocarcinoma [READ]) and gliomas (LGG and GBM) (Figure 3A). Although not fully understood, the lack of correlation may be dictated, in part, by the cell-of-origin, EMT states marked by distinct transcriptomic and epigenomic signatures [80] and unique mutational landscapes that collectively influence tumor cell phenotype during the course of malignant progression [81].

There is a growing body of evidence suggesting that CD73 participates in metastasis. Stagg et al. demonstrated that tumor-derived CD73 acts as a driver of metastasis in a murine model of breast cancer [52]. Furthermore, CD73-derived adenosine contributed to metastatic spread via promoting immune escape by targeting lymphocytes for immunosuppression while concomitantly enhancing the migratory capacity of tumor cells. In human gastric cancer, overexpression of CD73 was correlated with poor prognosis [82]. Importantly, single sample gene set enrichment analysis of TCGA gastric cancer cohort also demonstrated that CD73 high group was enriched in genes involved in cytoskeleton remodeling, which was confirmed in gastric cancer cell lines overexpressing CD73. Mechanistically, CD73 exerts its prometastatic effects by regulating β-catenin-induced EMT. Furthermore, targeting of CD73 was effective in prevented gastric cancer metastasis in an experimental setting [82]. Robinson et al. [83] carried out a comprehensive immunogenomic profiling of 500 cancer patients with metastatic solid cancers from over 30 primary sites (abbreviated as the “MET500” cancer cohort) using DNA- and RNA-sequencing, which serves as an important data resource to mine gene networks and gene expression patterns associated with metastasis. Interestingly, metastatic tumors were shown to fall into two main categories: EMT-like associated with inflammation or a proliferative subtype associated with metabolic stress. Furthermore, gene expression data demonstrated that metastatic tumors, in general, are significantly dedifferentiated. CD73 gene expression across these different metastatic sites was highly heterogeneous, with the highest expression observed in tumors that metastasize to the brain, breast, and thyroid (Figure 3B). These data from the integrated analysis display a similar pattern with the surgically resected primary tumors in the TCGA pan-cancer cohort (Figure 1A), suggesting that the tissue-specific microenvironment may affect the expression of CD73 at the metastatic sites. A separate analysis of different secondary tumors metastasized from the same primary origin, which showed that despite the same origin, these secondary tumors have a dramatic difference in CD73 expression at distinct metastatic sites (Figure 3C). It is important to note that tumors consist of a heterogeneous mix of tumor and stromal (immune/mesenchymal) components and whether CD73 expression in the microenvironment of the seeded organ is directly or indirectly involved in the metastatic process has not been extensively explored. Together, these data imply that adopting a strategy in targeting CD73 in treating patients with metastatic solid tumors may be context-specific.

Of note, primary (Figure 1A,B) and secondary (metastases) brain tumors (Figure 3B,C) display relatively high expression of CD73 compared to other types of tumors, which may prioritize CD73-based targeted therapy for either primary or secondary brain tumors. In the brain, the primary source of eADO comes from enzymatic hydrolysis of adenosine triphosphate (ATP, eATP) mediated by ectonucleotidases including CD73 [84]. There is an extensive literature on the role of eADO acting via high-affinity P1 adenosine receptors found expressed on both glial cells and neurons carrying out a critical role in normal brain behavior involving synaptic plasticity and memory formation, glutamate release, as well as regulation of vascular tone [85]. However, excessive CD73-adenosinergic signaling following CNS (central nervous system) pathologies such as ischemia, trauma and cancer becomes maladaptive [86]. GBM is the most aggressive type of glioma derived from glial cells. Chemotherapy-induced death in glioma leads to a dump of eATP providing an important source of eADO following ectonucleotidase-mediated hydrolysis [87]. eADO induces proliferation and enhances invasion mediated by P1 receptors. However, the cellular source of eADO that may provide a feedforward loop that augments tumor growth in gliomas. As described for other tumors, CD73 is also expressed on host-derived immune cells in glioma. A recent study highlights CD73 as a novel target for GBM [88]. Goswami et al. analyzed five different cancer types (GBM, non-small cell lung carcinoma, renal clear cell carcinoma, colorectal and prostate cancer; *n* = 94) that demonstrate both good and poor response to immune checkpoint inhibitors (ICIs) using mass cytometry and single-cell RNA sequencing. Intriguingly, they identified a unique population of blood-derived CD73^high^ macrophages enriched in an immunosuppressive gene signature in GBM patients. Analysis of the TCGA GBM cohort revealed an association between CD73^high^ macrophage gene signature and poor survival, and this population of cells was subsequently found to persist in a subset of patients with recurrent GBM treated with anti-PD-1 inhibitor pembrolizumab (NCT02337686). Furthermore, Goswami et al. showed that the absence of CD73 improved the efficacy of combined treatment with antibodies targeting immune checkpoint proteins cytotoxic T-lymphocyte-associated antigen 4 (CTLA-4) and PD-1, resulting in prolonged survival in a murine model of GBM [88]. Given the strong evidence showing the importance of CD73 in GBM, pathway enrichment analysis based on genes positively correlated with *NT5E* mRNA level in the TCGA GBM cohort may provide useful insight into unique biological mechanisms. Using this approach showed that the positively correlated genes are significantly enriched for pathways involved in focal adhesion, receptor tyrosine kinases signaling, extracellular matrix organization, antigen presentation: class I MHC, and interleukin−37 signaling (Figure 3D). In contrast, genes negatively correlated with *NT5E* mRNA level are mainly enriched for neuronal biological processes (Figure 3D), suggestive of a dedifferentiated state in CD73^high^ GBM. Collectively, this evidence suggests both cancer and host-derived cells cooperate to promote tumor immune evasion and combinatorial targeting may be necessary to improve antitumor immune responses to immune checkpoint therapy for GBM patients.

The above evidence reveals a close link between CD73 and EMT/stemness in cancer. This is not surprising since carcinoma cells responding to signals such as inflammation, hypoxia and TGFβ1, upregulate a transcriptional program that triggers EMT and stemness [17] also serve as drivers of CD73 expression. What is not as clear is whether CD73 functions as a cause or consequence of EMT/stemness in cancer. Recently, Weinberg and colleagues [89] demonstrated that activation of EMT program in a subset of breast carcinoma cells identified as quasi-mesenchymal Snail^HI^ overexpressed CD73, which protected epithelial carcinoma cells from immune destruction. Genetic inactivation of tumoral CD73 in quasi-mesenchymal Snail^HI^ breast carcinoma cells was sufficient to sensitize tumor cells to immunotherapy during metastatic breast cancer colonization. Importantly, using Chip-Sequencing, the authors were able to show that the EMT transcription factor Snail binds within the transcriptional start site within the CD73 promoter region positively regulating its expression. Thus, CD73 seems to provide the mechanistic link between EMT and cancer immune escape in the setting of breast cancer. This work builds upon previous work from Weinberg and colleagues showing that induction of this hybrid E/M state generates breast cancer cells with stem cell properties [90] and is essential for breast cancer tumorigenicity [91]. Interestingly, ectopic expression of the transcription factors Slug and Sox9 were used to drive this hybrid EMT cell state (Snail^HI^), which was previously shown to induce a stem cell state in breast carcinoma cells promoting tumorigenesis and metastatic seeding [92]. In a recent study, Lu and colleagues [50] demonstrated that CD73 exerts its pro-tumorigenic effects in liver cancer by regulating the stability of the transcription factor SOX9 contributing to stemness, tumor initiation and increased resistance. Moreover, CD73 working via enzymatic generation of adenosine was important for stemness and tumor initiation capacity in high-grade serous ovarian cancer [51], confirming results of an earlier study in patients with high-grade serous ovarian cancer where tumoral expression of CD73 was associated with poor survival and was strongly correlated with an EMT gene signature [20]. In these cases, CD73 acts as a driver of carcinogenesis; however, the relationship between EMT and stemness was not thoroughly investigated. Whether the EMT-stemness-CD73 link operates in a similar manner across primary human carcinomas also awaits further investigation. What also remains unclear is how the expression of CD73 enables metastatic founder cells to adapt to their microenvironment within foreign tissue (Figure 3C). Based on the role of CD73 in primary tumors, reshaping the foreign tissue tumor microenvironment by promoting immune suppression through enzymatic production of ADO and purinergic signaling might represent a likely culprit, although other mechanisms cannot be ruled out and await further investigation.

## 4. CD73 and Tumor Immune Microenvironment

Although cancer has primarily been studied as a cell-intrinsic disease, it is widely accepted that the tumor microenvironment (TME) plays an essential role in regulating plasticity. Along these lines, Malta et al. demonstrated that tumor types with higher stemness indices are correlated with reduced immune infiltration and PD-L1 expression at the protein level [62]. CD73 is an emerging immune checkpoint in modulating cancer progression via conversion of immunostimulatory eATP into immunosuppressive eADO [93,94]. As described above, CD73 nucleotidase activity promoting an immunosuppressive TME represents an ideal target to enhance immunotherapies in cancer, which to date are underwhelming in the majority of patients [71,95,96].

**Figure 4:**
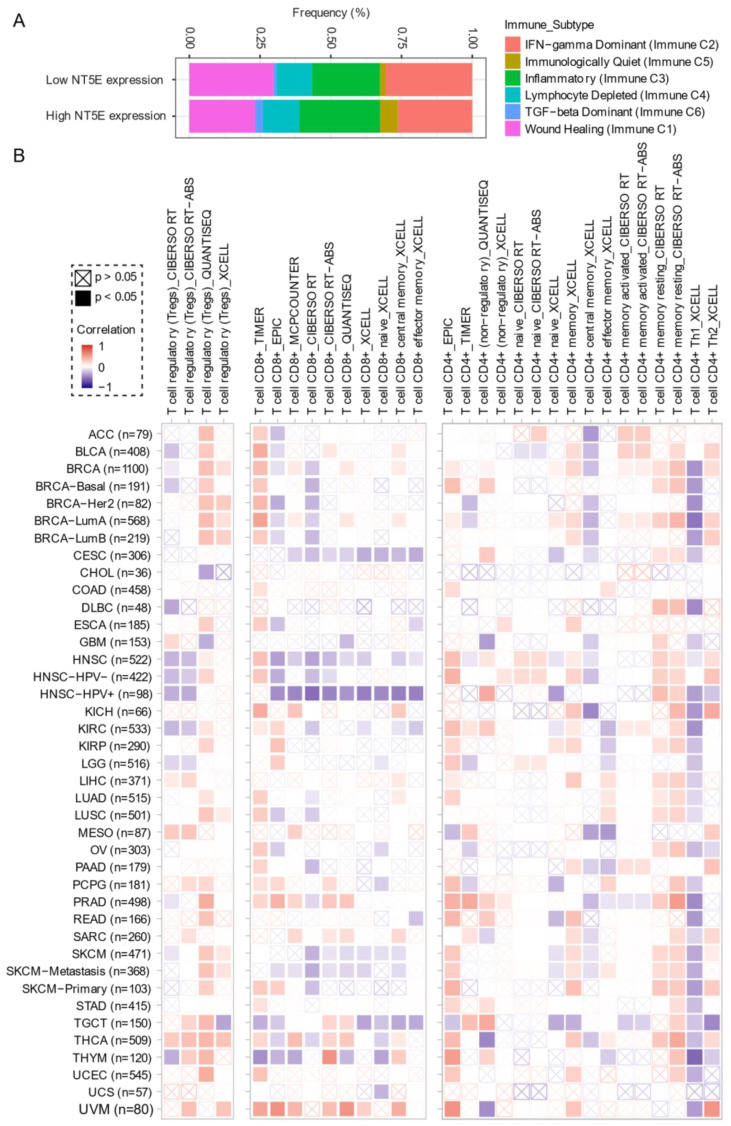
CD73 and tumor immune microenvironment. (**A**) Percentage (*NT5E* expression low vs. high) of immune subtype models (C1–C6) across TCGA (The Cancer Genome Atlas) pan-cancer cohort. The genes contained in each signature were evaluated using model-based clustering by *p* the “mclust” R package. Each sample was finally to be grouped based on its predominance with the C1–C6 signature. The immune subtype models were based on Thorsson V et al. Immunity. 2018 (see the methods in Appendix A). (**B**) Systematic correlation analysis of immune infiltrates (Tregs [left], CD8+ [middle], CD4+ [right]) with gene expression of *NT5E* across TCGA pan-cancer cohort. The number of patients was shown in parenthesis. Data were downloaded from TIMER (version 2.0), a comprehensive resource for systematic analysis of immune infiltrates across diverse cancer types (http://timer.comp-genomics.org/) (Ref. [98]). The red color indicates a positive correlation, while the blue color represents a negative correlation. The detailed information about the bioinformatic analysis can be found in Appendix A.

Comprehensive profiling of over 10,000 tumors across 33 diverse cancer types from TCGA pan-solid cancer cohort using a multi-omics approach uncovered six (C1–C6) immune subtypes [97]. This work provides a key resource for understanding tumor-immune interactions. The lymphocyte-depleted (immune C4) and TGF-beta dominant (immune C6) subtypes were marked by the worst prognosis and their immune makeup was consistent with an immunosuppressed TME. Accumulating evidence points towards CD73 shaping the TME [15,16]. Further exploration revealed that the Immunologically Quiet (Immune C5) and TGF−beta dominant (immune C6) immune subtypes are highly enriched in tumors with high *NT5E* expression compared with low *NT5E* expression (Figure 4A). To gain a clearer picture of the immune infiltrates across the tumor subtypes which may be of potential relevance to cancer immunology, we utilized TIMER, an algorithm that provides information regarding proportions of immune cell types by multiple immune deconvolution methods [98]. Focusing primarily on T cells, we demonstrate that T cell regulatory (Tregs)_QUANTISEQ fraction is positively correlated with CD73 gene expression across a wide number of tumor types including BRCA and THCA. In addition, CD73 gene expression displays a broadly positive correlation with the T cell CD8+_TIMER and multiple CD4+ fractions (Figure 4B). This pan-cancer dataset suggests a mixture of suppressive and activated immune infiltrates together with high CD73 gene expression. For example, in HNSC a negative correlation between CD73 and CD8+ T cell infiltration, including HPV-positive patients was observed (Figure 4B). This observation supports a recent study from Watermann et al. [99] demonstrating that recurrent HNSC has an immunosuppressive tumor microenvironment with significant depletion of CD8 tumor-infiltrating lymphocytes (TILs), which was more pronounced following adjuvant chemo-radiotherapy. Therefore, these tumors might be less susceptible to respond to ICIs due to the lack of infiltrating CD8+ CTLs. Whether anti-CD73 treatment can turn an immunosuppressive cold tumor-like HNSC into a hot tumor awaits further investigation. It is important to note that CD73-mediated production of immunosuppressive adenosine has also been described for tumor-infiltrating B cells [100] and NK cells [101] whereby tumors can hijack both B cell- and NK cell-mediated suppression of activated T cells to escape immunity.

## 5. CD73 and Therapy Resistance

One of the main goals in cancer research is to elucidate the molecular mechanisms of resistance to therapy. Besides a genetic basis, it is becoming increasingly appreciated that to win the fight against cancer, beyond the development of strategies aimed at killing cancer cells, it also will be important to stimulate the immune response to keep the residual tumor cells in check. Evidence has shown increased CD73 protein expression in cancer cells developing resistance to various therapies, e.g., chemotherapy [55,102], radiotherapy [56,103], and targeted therapy [104,105], as well as immunotherapy [71,96], suggesting that this enzyme is involved in treatment resistance. However, CD73 is also expressed on host-derived cells found within the TME such as immunosuppressive Tregs, B cells, NK cells, macrophages and CAFs. In this regard, therapeutic resistance involves both intrinsic and extrinsic mechanisms. The effect of targeting CD73 is likely to be mediated by eradicating the immunosuppressive and proangiogenic niche within the TME that is regulated by CD73, as stimulating the immune response can facilitate the elimination of the residual cancer cells. As such, targeting both tumor- and host-derived CD73 could represent a novel way to increase the efficacy of a diversity of antineoplastic treatments (i.e., chemotherapy, targeted therapy, and immunotherapy).

### 5.1. Chemotherapy

Emerging evidence has demonstrated that the antitumor effect of traditional chemotherapy is not only related to its inhibition of DNA replication and/or induction of DNA damage but also from eliciting activation of the host immune system due to immunogenic cell death following the presentation of neo-epitopes from dead and dying tumor cells. While immunogenic cell death is critical for the effectiveness of chemotherapy [106], the death of cancer cells also results in the release of ATP eventually being converted to immunosuppressive eADO within the TME following ectoenzymatic breakdown mediated by CD73 [87]. Therefore, chemotherapy can act as a two-edged sword.

The association of CD73 and chemotherapy resistance has been mostly investigated in breast cancer [25,107,108,109,110]. High CD73 expression is associated with poor prognosis and therapeutic response rate to chemotherapy in TNBC [109,110], a highly aggressive cancer type that lacks effective treatment strategies. Recently, Loi, et al. reported that CD73 in tumor cells conferred the resistance to doxorubicin, through suppressing adaptive antitumor immune responses by activating high-affinity A2A adenosine receptors [110]. Thus, targeting CD73 enhanced doxorubicin-mediated antitumor immune responses and significantly prolonged the survival of mice with metastatic breast cancer. A more recent study by Samanta et al. [25] demonstrated chemotherapy (carboplatin, doxorubicin, gemcitabine, or paclitaxel) promotes the enrichment of an immune evasive subpopulation of TNBC marked by co-expression of CD47/CD73/PD-L1 transcriptionally regulated via HIF1α. The cell surface protein CD47, a myeloid-specific immune checkpoint molecule, binds its cognate ligand SIRPα (signal regulatory protein α) on macrophages inducing anti-phagocytosis protecting tumor cells, whereas CD73 and PD-L1 suppress cytotoxic CD8+ TILs through independent mechanisms.

Along the same lines, Buisseret et al. [108] reported the clinical significance of CD73 for TNBC patients based on the Breast International Group (BIG) 02-98 adjuvant prospective phase III clinical trial that compared the addition of docetaxel to doxorubicin with doxorubicin-based chemotherapy in node-positive breast cancers. Based on multiplex immunofluorescence and image analysis, they quantitatively assessed CD73 expression on tumor cells, tumor-infiltrating leukocytes and stromal cells, which showed a higher expression of CD73 on tumor and immune cells compared to stromal cells. Moreover, higher CD73 expression of tumor and immune cells was observed in patients with significant (>10) lymph node invasion. Additionally, increased CD73 expression on tumor cells but not on stromal and immune cells was correlated with poor survival. More importantly, utilization of CD73 expression on tumor cells together with tumor immune infiltration degree allowed the authors to identify subgroups of patients with a distinct prognosis, with the worst prognosis in TNBC patients with high CD73 expression and low immune infiltration. Reinforcing the prognostic importance of CD73 in human breast cancer, Hu et al. (2020) reported an enrichment in CD73+ γδTregs in treatment-naïve breast cancer patients that correlated with worse overall survival in TNBC, HER2+ and luminal subtypes [111]. Importantly, CD73+ γδTregs and CAFs promoted tumor progression via the formation of an IL6-eADO positive feedback loop targeting infiltrating CD8+ CTLs for immunosuppression. The association between CD73 and the acquired resistance to chemotherapeutics has also been described in several other cancer types, including solid [40,67,112,113,114] and blood [115] cancers. Collectively, these lines of evidence support CD73 as a promising treatment to enhance the efficacy of chemotherapy in cancers and potentially tumor immunogenic cold tumors into hot tumors.

Despite the critical role of CD73 in chemotherapy resistance, several questions remain. For instance, the molecular mechanism(s) whereby anti-CD73 therapy sensitizes tumor cells to chemotherapy is not clear. Recently, Qiao et al. [116] reported that anti-CD73 antibody inhibits cell migration and invasion in both human TNBC and mouse 4T1 cell lines, although anti-CD73 antibody alone did not affect tumor growth. Mechanistically, anti-CD73 treatment was shown to activate autophagy in tumor cells blocking their tumor cell migration and invasion [116]. Moreover, the mechanisms whereby CD73 promotes chemotherapy resistance in cancer cells treated with DNA-damaging reagents have not been fully understood. Nicotinamide adenine dinucleotide (NAD+), as a substrate for the consuming enzymes such as PARPs and sirtuins, is involved in diverse biological processes, e.g., cell metabolism, DNA repair, and genomic stability [117]. Previous evidence showed that CD73 regulates intracellular NAD+ levels by processing NAD+ and its bio-precursor, nicotinamide mononucleotide (NMN), which may play a role in enhancing DNA repair capacity thereby promoting chemotherapy resistance [118,119,120,121]. As such, co-targeting CD73 was shown to potentiate nicotinamide phosphoribosyltransferase (NAMPT), the rate-limiting enzyme in NAD+ biosynthesis, in a murine model of ovarian cancer [120]. However, Wilks et al. [122] recently demonstrated that extracellular NAD+ enhances PARP-dependent DNA repair capacity, but the effect is independent of CD73 activity. Further studies are warranted to understand whether and how CD73 blockage can enhance chemosensitivity and inhibit tumor metastases in immune-free in vitro and immune-competent in vivo models.

### 5.2. Targeted Therapy

Infiltration of tumors by cytotoxic T cells is not only associated with improved response to chemo-radiation, but also contributes to the effects of targeted therapies. Accumulating evidence demonstrates that targeting CD73 can enhance the effect of inhibitors targeting the EGFR (epidermal growth factor receptor) family in immune-competent preclinical mouse models.

A study by Turcotte et al. showed that CD73 promotes the resistance to HER2/ErbB2 (human epidermal growth factor receptor 2)-based targeted therapy in breast cancer [104]. Specifically, in HER2/ErbB2-driven breast cancer, CD73 expression by tumor cells and host cells significantly suppressed immune-mediated responses mediated by the anti-ErbB2 blockage. CD73 blockade enhances the anti-tumor activity of the anti-ErbB2 antibody against engrafted or spontaneous tumors, as well as lung metastases. Further, gene ontology enrichment analysis from gene-expression data revealed a positive association between CD73 expression and extracellular matrix organization, TGF-β genes, EMT process and HIF-1 gene signature. This study highlights the importance of targeting CD73 to potentiate HER2/ErbB2-targeted therapy in breast cancer.

Likewise, immune metabolic reprogramming mediated by CD73-adenosine signaling has also been shown critical in the context of NSCLC (non-small cell lung cancer) harboring EGFR mutations, the presence of which typically predicts unresponsiveness to immunotherapy and is generally used as a biomarker to exclude patients for ICIs. Evidence shows that *EGFR*-mutation drives a low immunogenic tumor microenvironment characterized by a lack of TILs and a low mutation burden [123]. However, the biological mechanisms underlying the immune escape in *EGFR*-mutant NSCLC is not clear. Interestingly, a relationship between CD73-shaped suppressive immune microenvironment and *EGFR* mutation in tumors has been revealed. CD73 expression is significantly increased together with decreased tumor necrosis factor (TNF) expression in NSCLC samples harboring *EGFR* mutations compared with wild-type tumors [19]. Moreover, CD73 can also reciprocally regulate EGFR expression in breast cancer [107]. In NSCLC patients with high PD-L1 expression (>50%), EGFR-TKI targeted therapy-induced an increased expression of CD73 in tumor cells compared to baseline and might explain, in part, the poor efficacy obtained in some patients in response to ICI targeting programmed death 1 (PD1) on T cells [124]. In a separate study, high compared with low CD73 expression predicted a favorable response to ICIs in patients with advanced or recurrent *EGFR*-mutant NSCLC. In contrast, CD73 expression had no impact on response rates to ICIs in *EGFR* wild-type patients [125]. These lines of evidence highlight the role of CD73 in shaping suppressive immune microenvironment specific to *EGFR* mutations, which may explain in part, the limited responsiveness to immunotherapy in *EGFR*-mutant NSCLC. Despite this, the exact relevance of CD73-adenosine signaling in *EGFR*-mutant NSCLC to the efficacy of ICIs remains unclear. Recently, Tu et al. [126] demonstrated that combined anti-PD-L1 and anti-CD73 therapy promoted T cell response to *EGFR*-mutant NSCLC and induced more tumor shrinkage compared with anti-PD-L1 or anti-CD73 treatment alone. At the molecular level, combination therapy dramatically increased the number of MART1-specific CD8+ T cells in the tumor and frequency of memory precursor CD62L+/CD45RO+/ CCR7+CD8+ T cells in the spleen, which coincided with enhanced IFN-γ production by tumor antigen-specific CD8+ T cells. The evidence by Tu et al. suggests that targeting CD73 may help to reinvigorate the host immune system, in particular cancer patient-specific CD8+ CTLs, providing the rationale for an ongoing clinical trial investigating combined anti-CD73 and anti-PD-L1/PD-1 therapy for NSCLC patients with *EGFR* mutation (NCT03381274).

The discovery of immune checkpoint proteins including but not limited to PD1 protein and its ligand PD-L1, as well as CTLA-4, has revolutionized cancer treatment [127]. Engagement of PD1 by PD-L1 acts as a natural break for the immune system during periods of heightened inflammation, and tumors hijack PD-L1 as a means to escape immune recognition. Treatment of patients with monoclonal antibodies targeting immune checkpoint proteins prolongs survival across several cancers including melanoma and NSCLC. ICIs are thought to target the tumor cell-immune crosstalk and reinvigorate tumor antigen-specific lymphocytes. Despite these positive responses, the majority of patients fail to respond to ICIs and some patients progress while on immunotherapy and even acquire resistance [128].

In the setting of melanoma [33], CD73-adenosinergic signaling regulates melanoma phenotypes in response to stress in the form of mitogenic, inflammatory and hypoxic signals. In subsets of melanoma patients progressing after immunotherapy with dedifferentiated tumors showed an upregulation of CD73 compared with pre-treatment suggestive of an immune adaptive resistance mechanism. In this setting, CD73 might serve as a novel biomarker to stratify patients with melanoma for ICIs.

## 6. CD73 and Drug Repurposing

The above evidence highlights the importance of blocking the CD73-adenosinergic signaling pathway in the treatment of cancer. Currently, there is an intense effort underway developing novel monoclonal antibodies or selective small molecules targeting both CD73 or P1 adenosinergic receptors (A2aRA, A2aRB) for cancer and we refer the reader to recent in-depth reviews covering this [16,129]. Despite the promise of CD73 as a therapeutic target for cancer treatment, there are currently no clinically approved CD73 inhibitors for the treatment of cancer. One of the main reasons behind this is the lack of biomarkers of CD73-adenosine rich tumors to guide precise clinical management, which may lead to heterogeneous treatment responses and rapid development of resistance to CD73 targeted therapy. Preclinical mouse models and human studies confirm the link between hypoxia and CD73-adenosinergic rich tumors and chemoresistance [130]. Therefore, a bioinformatics biomarker-guided stratification that will identify patients most likely to benefit from CD73 targeted therapy alone or in combination with chemotherapy, targeted, or immunotherapy is needed.

**Figure 5:**
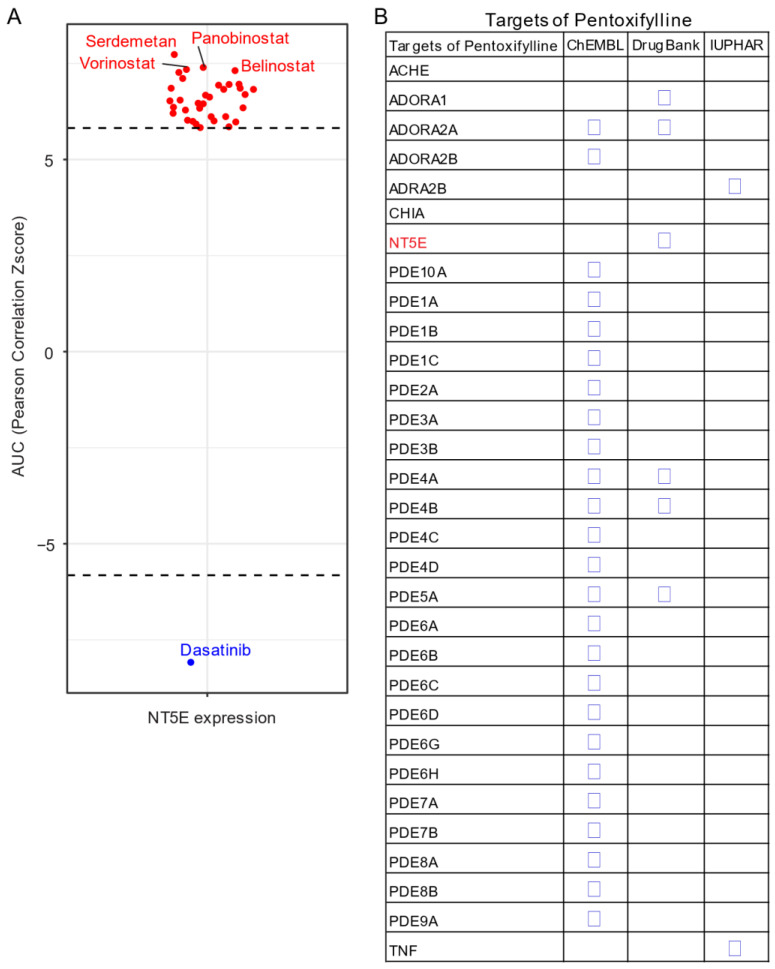
Drug repurposing for CD73 targeted therapy. (**A**) Integrated correlation analysis of *NT5E* gene expression with drug (*n* = 481) response profiles (reflected by Z-score normalized area under the curve [AUC] value) across solid cancer cell lines (*n* = 659). Red dots indicate drugs whose AUC value significantly (adj. *p* < 0.05) positively correlates with *NT5E* gene expression, while the blue represent the significantly negatively correlated ones. The drug response data were downloaded from a previously published study (see the methods in Appendix A). (**B**) Drug repurposing identifying *NT5E* as one target of Pentoxifylline. Data were downloaded from ReframeDB database (https://reframedb.org/). The detailed information about the bioinformatic analysis can be found in Appendix A.

Given the unavailability of clinically approved CD73 inhibitors for cancer, drug repurposing, which is intended to find new uses for clinically approved drugs [131], may hold promise as an alternative and cost-effective way of targeting the CD73-adenosinergic signaling pathway. The advantage of this approach is that the existing pharmacokinetic/pharmacodynamic, as well as safety profiles have already been established [131]. To systematically investigate potential inhibitor candidates that can modulate the efficacy of CD73 inhibitors, drug sensitivity profiles of hundreds of compounds (n = 481) were correlated with CD73 mRNA level across a panel of solid cancer cell lines (n = 659) [132]. Of note, a negative correlation means that cancer cells with a high expression of CD73 have a lower AUC (area under the curve) value in response to the indicated inhibitors representing a sensitive drug compound (Figure 5A). In contrast, a positive correlation indicates that solid cancer cells with a high expression of CD73 are more resistant to the corresponding drug compounds. Intriguingly, we observed that dasatinib (brand name Sprycel), a clinically approved inhibitor selectively targeting BCR-ABL/SRC used in the treatment of chronic myeloid leukemia and acute lymphoblastic leukemia, is the only compound whose AUC value significantly negatively correlates with the expression of CD73. Conversely, several pan-HDAC inhibitors appear as the top whose AUC significantly positively correlated drug compounds. Therefore, cancer cells with enhanced CD73 activity might be sensitive to dasatinib treatment, but resistant to pan-HDAC inhibitors. Regarding this, we and others have previously shown that dasatinib not only inhibits cancer cells but also modulates the tumor immune microenvironment and enhances the efficacy of ICIs [133,134,135]. Others have shown that dasatinib effectively blocks TGFβ-induced expression of transcription factors promoting EMT and may be a novel therapeutic option in pancreatic and prostate cancer [136] and pulmonary sarcomatoid carcinoma, a rare and deadly form of NSCLC [137].

Based on a public drug repurposing database ReframeDB (https://reframedb.org/), we have found that the synthetic dimethylxanthine derivative pentoxifylline (PTX), clinically approved for the treatment of peripheral vascular diseases and osteoradionecrosis, as well as the management of cerebrovascular insufficiency [138,139] also targets the CD73-adenosinergic signaling pathway (Figure 5B). Originally identified as a nonselective phosphodiesterase inhibitor (PDE4B, PDE4A, PDE5A), PTX also has additional anti-inflammatory, immunomodulatory and bronchodilatory effects due to its ability to act as a non-selective adenosine receptor antagonist (A1, ADORA1 and A2a, ADORA2A) and *NT5E*/CD73 inhibitor [140]. CD73 is upregulated following radiotherapy and is linked to radiation-induced tissue injury [141] and PTX shows protective effects against radiotherapy-induced lung toxicity in both breast and lung cancer [142]. Blocking radiotherapy-induced CD73 upregulation promotes host-mediated immune rejection of tumors [143]. Collectively, repurposing of well-known clinically approved drugs targeting CD73-adenosine together in combination with dasatinib and/or PTX with ICIs may represent an ideal combination therapy in solid tumors with an extensive desmoplastic stroma, which warrants further investigation.

## 7. Conclusions

Overall, these translational lines of evidence highlight a critical role of CD73 in engaging tumor plasticity. Further studies will be needed to determine the underlying molecular mechanisms, which to date remain largely unknown. Moreover, there is a real potential for drug repurposing using dasatinib together with PTX in the treatment of CD73-adenosine rich advanced primary and metastatic tumors.

## Data Availability

No new data were created or analyzed in this study. Data sharing is not applicable to this article.

## Appendix A

Methods: bioinformatic analysis and public databases.

### Appendix A.1. TCGA Pan-Cancer Dataset

Transcriptomic and genomic landscape data of TCGA (The Cancer Genome Atlas Program) pan-cancer cohort were downloaded from cBioPortal (https://www.cbioportal.org/), and only primary tumor samples and the matched normal tissue were included.

#### Survival Analysis

Forest blots showing the survival analysis of cancer patients stratified by the gene expression of NT5E across the TCGA pan-solid cancer cohort. The “high” and “low” expression groups were stratified by the optimal cutoff value using “survminer” and “survival” packages in R software (version 3.6.3) [133,144,145]. *p* < 0.05 was considered statistically significant.

### Appendix A.2. Stemness and EMT Signature Score

The curated mRNA- and epigenetics-based Stemness Scores [62] derived by Stemness group was used and downloaded from UCSC Xena [146]. Curated epithelial-to-mesenchymal transition (EMT) scored as the sum of a mesenchymal gene set (*FN1 + VIM + ZEB1 + ZEB2 + TWIST1 + TWIST2 + SNAI1 + SNAI2 + CDH2) minus that of epithelial genes (CLDN4 + CLDN7 + TJP3 + MUC1 + CDH1*) [78,79].

### Appendix A.3. MET500 Metastatic Tumor Cohort

Data for the *NT5E* gene expression across the metastatic tumors at different biopsy sites were downloaded from the MET500 cancer cohort, which provides the transcriptomic data of 500 adult patients with various metastatic solid tumors [83]. The normalized gene expression of NT5E was extracted.

### Appendix A.4. KEGG and Reactome Pathway Enrichment Analysis

The genes whose expression significantly correlate with NT5E in TCGA GBM were selected to explore their biological functions, such as Kyoto Encyclopedia of Genes and Genomes (KEGG) and Reactome pathways, using the R package “clusterprofiler” [147,148]. Cnetplot was used to list gene names of the proteins enriched in the GO pathway.

### Appendix A.5. Tumor Immune Infiltrates

Tumor-infiltrating immune cell profiles across TCGA pan-cancer cohort were downloaded from TIMER (version 2.0), a comprehensive resource for systematic analysis of immune infiltrates across diverse cancer types (http://timer.comp-genomics.org/) [98]. Curated immune subtype models (C1–C6) were based on a previous study [97]. The genes contained in each signature were evaluated using model-based clustering by *p* the “mclust” R package. Each sample was finally to be grouped based on its predominance with the C1–C6 signature.

### Appendix A.6. Drug Repurposing Databases

Data for the integrated correlation analysis of NT5E gene expression with drug (*n* = 481) response profiles (reflected by Z-score normalized area under the curve [AUC] value) across solid cancer cell lines (*n* = 659) were downloaded from a previous study [132].

Data for the drug repurposing identifying NT5E as one target of Pentoxifylline were downloaded from a publicly curated database (https://clue.io/command).
